# Alkaline phosphatase for treatment of sepsis-induced acute kidney injury: a prospective randomized double-blind placebo-controlled trial

**DOI:** 10.1186/cc11159

**Published:** 2012-01-23

**Authors:** Peter Pickkers, Suzanne Heemskerk, Jeroen Schouten, Pierre-François Laterre, Jean-Louis Vincent, Albertus Beishuizen, Philippe G Jorens, Herbert Spapen, Michael Bulitta, Wilbert HM Peters, Johannes G van der Hoeven

**Affiliations:** 1 Department of Intensive Care, Nijmegen Institute for Infection, Inflammation and Immunity, Radboud University Nijmegen Medical Center, Geert Grooteplein Zuid 10, Nijmegen, 6525 GA, The Netherlands; 2 Department of Pharmacology and Toxicology, Institute for Genetic and Metabolic Disease, Geert Grooteplein Noord 21, Nijmegen, 6525 EZ, Radboud University Nijmegen Medical Center, The Netherlands; 3 Department of Critical Care Medicine, Canisius Wilhelmina Ziekenhuis, Weg door Jonkerbos 100, Nijmegen, 6532 SZ, The Netherlands; 4 Department of Intensive Care Medicine, Cliniques Universitaires Saint Luc-UCL, Avenue Hippocate 10, Brussels, 1200, Belgium; 5 Department of Intensive Care Medicine, Hopital Erasme, Université Libre de Bruxelles, Route de Lennik 808, Brussels, 1070, Belgium; 6 Department of Intensive Care Medicine, VU University Medical Center, De Boelelaan 1117, Amsterdam, 1007 MB, The Netherlands; 7 Department of Intensive Care Medicine, University Medical Center Antwerp, Wilrijkstraat 10, Edegem, 2650, Belgium; 8 Department of Intensive Care Medicine, University Hospital VUB, Laarbeeklaan 101, 1090, Jette, Belgium; 9 CRM Biometrics GmbH, Weiherstrasse 19, Rheinbach, 53359, Germany; 10 Department of Gastroenterology, Radboud University Nijmegen Medical Center, Geert Grooteplein Zuid 8, Nijmegen, 6525 GA, The Netherlands

**Keywords:** sepsis, systemic inflammatory response syndrome, septic shock, acute renal failure, therapy

## Abstract

**Introduction:**

To evaluate whether alkaline phosphatase (AP) treatment improves renal function in sepsis-induced acute kidney injury (AKI), a prospective, double-blind, randomized, placebo-controlled study in critically ill patients with severe sepsis or septic shock with evidence of AKI was performed.

**Methods:**

Thirty-six adult patients with severe sepsis or septic shock according to Systemic Inflammatory Response Syndrome criteria and renal injury defined according to the AKI Network criteria were included. Dialysis intervention was standardized according to Acute Dialysis Quality Initiative consensus. Intravenous infusion of alkaline phosphatase (bolus injection of 67.5 U/kg body weight followed by continuous infusion of 132.5 U/kg/24 h for 48 hours, or placebo) starting within 48 hours of AKI onset and followed up to 28 days post-treatment. The primary outcome variable was progress in renal function variables (endogenous creatinine clearance, requirement and duration of renal replacement therapy, RRT) after 28 days. The secondary outcome variables included changes in circulating inflammatory mediators, urinary excretion of biomarkers of tubular injury, and safety.

**Results:**

There was a significant (*P *= 0.02) difference in favor of AP treatment relative to controls for the primary outcome variable. Individual renal parameters showed that endogenous creatinine clearance (baseline to Day 28) was significantly higher in the treated group relative to placebo (from 50 ± 27 to 108 ± 73 mL/minute (mean ± SEM) for the AP group; and from 40 ± 37 to 65 ± 30 mL/minute for placebo; *P *= 0.01). Reductions in RRT requirement and duration did not reach significance. The results in renal parameters were supported by significantly more pronounced reductions in the systemic markers C-reactive protein, Interleukin-6, LPS-binding protein and in the urinary excretion of Kidney Injury Molecule-1 and Interleukin-18 in AP-treated patients relative to placebo. The Drug Safety Monitoring Board did not raise any issues throughout the trial.

**Conclusions:**

The improvements in renal function suggest alkaline phosphatase is a promising new treatment for patients with severe sepsis or septic shock with AKI.

**Trial Registration:**

www.clinicaltrials.gov: NCTNCT00511186

## Introduction

Septic shock is the most common cause of acute kidney injury (AKI) [1], and is associated with considerable morbidity and mortality [2-4]. Currently, there is no single drug approved for the treatment of sepsis-induced AKI [5].

Alkaline phosphatase (AP) is an endogenous enzyme that exerts detoxifying effects through dephosphorylation of endotoxins [6,7] and pro-inflammatory extracellular ATP [8]. Local AP concentrations reflect the host defense against endotoxin in the kidney [9], and during ischemia enzyme levels are markedly depleted, associated with the development of AKI [10]. Apart from local effects in the kidney, AP may attenuate the innate immune response, as dephosphorylation of endotoxin abolishes its biological activity and induces tolerance to subsequent endotoxin exposure [11]. In animal models of sepsis, AP administration attenuates the inflammatory response and reduces mortality [12,13].

In a previous trial investigating the safety and pharmacokinetics in patients with sepsis (with and without evidence of AKI), Heemskerk *et al*. showed that administration of AP was associated with a decreased expression of inducible nitric oxide synthase in proximal tubule cells isolated from urine related to an attenuated urinary excretion of the proximal tubule injury marker glutathione S-transferase A1-1 (GSTA1-1) [14]. However, this previous trial had not been powered to test clinical renal endpoints, as it had also enrolled patients without AKI. Therefore, the current prospective trial focusing on severe sepsis patients or septic shock with evidence of AKI was required to examine the effects of AP on clinical renal endpoints, urinary excretion of various markers of tubular injury, and circulating inflammatory markers.

## Materials and methods

### Patient selection and design

All patients who were admitted to one of the seven participating intensive care units (ICUs, three Dutch, four Belgian) were enrolled from June 2008 to November 2009 after approval by the hospitals' Ethics Committees, national authorities and trial registration (NCT 00511186). Following written informed consent from the subject or legal representative, patients with severe sepsis or septic shock [15] and AKI who fulfilled the protocol selection criteria (Table 1) were randomized in a double-blind, placebo-controlled design. Treatment consisted of an intravenous bolus injection of AP (calf intestinal AP; AM-Pharma, Bunnik, The Netherlands) 67.5 U/kg bodyweight over 10 minutes followed by continuous infusion of 132.5 U/kg/24 h followed-up for 28 days post-entry.

**Table 1:** Patient entry selection

Inclusion criteria
• Age: 18 to 80 years, inclusive
• Diagnosis: proven or suspected infection
• Two out of four SIRS criteria of systemic inflammation [36]
- Core temperature > 38° or < 36° Celsius
- Heart rate > 90 beats/minute (unless the patient has a medical condition known to increase heart rate or is receiving treatment that would prevent tachycardia)
- Respiratory rate > 20 breaths/minute, PaCO_2 _< 32 mmHg or the use of mechanical ventilation for an acute respiratory process
- White-cell count > 12,000/mm^3 ^or < 4,000/mm^3 ^or a differential count showing > 10% immature neutrophils.
• Acute Kidney Injury, defined as:
• Rise in serum creatinine level to > 150 μmol/L (1.70 mg/dL) within the previous 48 hours, in the absence of primary underlying renal disease, OR
• Minimally at Stage 1 Kidney Injury according to AKIN creatinine criteria: Increase in serum creatinine > 26.2 μmol/L (0.30 mg/dL) or increase to > 150% (> 1.5-fold) from baseline in the previous 48 hours in the absence of primary underlying renal disease and where baseline creatinine is less than 150 μmol/L (1.70 mg/dL)), OR
• Minimally at Stage 1 Kidney Injury (AKIN) Urine Output criteria: Urine Output < 0.5 mL/kg/h for > 6 h and following adequate fluid resuscitation when applicable, in the absence of underlying primary renal disease and where baseline creatinine is less than 150 μmol/L (1.70 mg/dL).
• Written informed consent obtained prior to any study intervention. In addition to the above, acute onset of end-organ dysfunction (other than renal failure) in the preceding 12 hours unrelated to the primary septic focus and not explained by any underlying chronic disease [15]may be present (not compulsory) for patient qualification for enrollment)
**Exclusion criteria**
• Pregnant women or nursing mothers and fecund females not on effective contraception
• Known HIV (sero-positive) patients
• Patients already on RRT at entry
• Patients receiving immunosuppressant therapy or on chronic high doses of steroids equivalent to prednisone 1 mg/kg/day
• Patients expected to have rapidly fatal disease within 24 hours
• Known confirmed gram-positive sepsis
• Known confirmed fungal sepsis
• Acute pancreatitis with no established source of infection
• Any previous administration of exogenous AP
• Participation in another investigational study within 90 days
• Patients not expected to survive for 28 days due to other medical conditions such as end-stage neoplasm
• Known allergy to dairy products including cow milk
• Sepsis without renal failure as defined in Entry Criteria
• History of chronic renal failure or history of persistent creatinine level equal or greater than 150 μmol/L (1.70 mg/dL) prior to entry for reasons other than the current sepsis condition

AP, alkaline phosphatase; PaCO_2_, partial arterial pressure of carbon dioxide; RRT, renal replacement therapy; SIRS, systemic inflammatory response syndrome.

Patients were randomly assigned in a 1:1 ratio to AP or placebo. Study medication was packaged according to a randomization list in blocks of four, labeled with subject number and additional administration information. Each patient to be entered in the study was assigned the next consecutive number. Both placebo and AP were supplied as a clear, colorless, sterile, pyrogen-free solution in 10 mL type I glass vials with a Teflon-coated bromobutyl rubber stopper and indistinguishable from each other. The responsible pharmacist calculated the exact volume of AP or placebo to be administered to each patient based on body weight and treatment code. The master randomization list was held by Choice Pharma Ltd. 65 Knowl Piece Wilbury Way, Hitchin Hertfordshire, SG4 0TY UK. Individual sealed envelopes with the treatment code for each patient were filed in a secure location at each hospital and at Choice Pharma Ltd. 65. Data were entered into a clinical database by CRM Biometrics (Boehringer Mannheim, Germany). AKI was defined according to the Acute Kidney Injury Network (AKIN) criteria [16] and the criteria for initiation of RRT (in all cases continuous veno-venous hemofiltration, CVVH) were standardized per the Acute Dialysis Quality Initiative (ADQI) consensus [17].

Apart from the safety endpoints required by regulatory authorities for phase-II trials, the primary efficacy outcome measure was a prospectively defined composite end-point, including recovery of endogenous creatinine clearance (eCrCl), the need for RRT throughout the 28-day study period, and the total duration (hours on RRT per total number of patients; cumulatively for multiple interventions over the period). eCrCl was measured every 24 h with the following formula: eCrCl (mL/minute) = (Urine Creatinine (μM or mg/dL) × Volume (mL))/(Serum Creatinine (μM or mg/dL) × Time (minutes)

In addition, combinations of alternative parameters were tested exploratorily (see Additional file 1).

The secondary endpoints were changes in the urinary excretion of biomarkers of renal injury [18,19]: kidney injury molecule-1 (KIM-1), neutrophil gelatinase-associated lipocalin (NGAL), interleukin-18 (IL-18), glutathione S-transferase (GST)A1-1 and GSTP1-1 (see additional file 2); serum concentrations of lipopolysaccharide-binding protein (LBP), IL-6, C-reactive protein (CRP), and procalcitonin (PCT); changes in Sequential Organ Failure Assessment (SOFA) score; duration of ventilator support; length of ICU and hospital stay; and all-cause mortality.

Safety was evaluated by adverse event (AE) monitoring and any abnormalities during clinical management throughout the trial, reported according to MedDRA (Medical Dictionary for Regulatory Activities [20]), and overseen by an independent Data Safety Monitoring Board (DSMB).

This trial was designed by P Pickkers and JG van der Hoeven and approved by AM-Pharma. All investigators were responsible for their own data collection. Statistical analysis was conducted by an independent agency: CRMB Biometrics GmbH, Boehringer Mannheim, Germany (J Schmitz and J Hartung). AM-Pharma was not involved in the interpretation of the data, in the preparation of the manuscript or in the decision to submit the manuscript for publication. The trial was funded by a grant from AM-Pharma BV who provided active and placebo AP enzyme.

### Assays methodology

Arterial blood (arterial line, every 12 h) and urine (indwelling catheter, every 6 h) were freshly collected (and subsequently frozen at -80°C) during 48 h of treatment and daily thereafter until Day 7. Urinary KIM-1, NGAL, IL-18, GSTA1-1 and GSTP1-1 [18,19] were assayed in duplicate by enzyme-linked immunosorbent assay (ELISA), maximum intra- and interassay coefficient of variation (CV) of 10 and 15%, respectively (see Additional file 2). Routine hematology, biochemistry and CRP were evaluated by each hospital's laboratory; PCT was analyzed centrally at UMC Laboratories (Nijmegen, The Netherlands); LBP and IL-6 were determined centrally by TNO Quality of Life Laboratories (Zeist, The Netherlands).

### Statistics

The intention-to-treat (ITT) population was used for efficacy evaluations, per prospective Statistical Plan. Since the variation of the combined clinical renal end-points in septic patients is unknown, the hypothesis assumed a standardized difference of 1.0 (mean treatment difference/SD), with 80% power and alpha at 5% (two-sided), which required at least 17 patients per treatment group. Appropriate methodology was applied to the data regarding testing for distribution, including application of central limit theorem [21] where parametric tests were applied to non-normally distributed data. The primary efficacy measure of combined renal parameters was calculated according to the method of Hartung [22]; eCrCl was analyzed by repeated measures ANOVA, RRT requirement by Fisher's exact test, and RRT duration by t-test. For eCrCl, missing values (typically after Day 7, due to ICU discharge) were imputed from last-observation-carried forward (LOCF). Systemic and urinary biomarkers were analyzed by repeated measures ANOVA with baseline concentrations as covariate. Safety variables were compared using Fisher's exact test. Analysis was performed using SAS version 9.2 (SAS Institute Inc, Cary, NC, USA).

## Results

**Figure 1:**
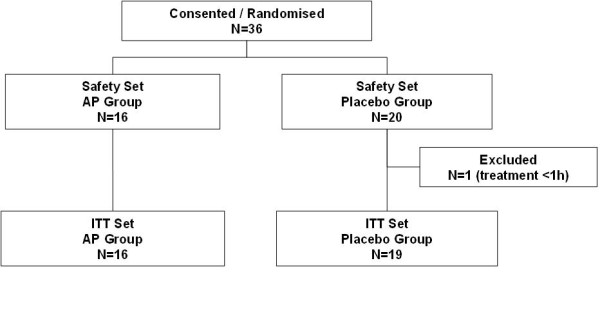
**Flow chart of patients**.

The two treatment groups (flow chart illustrated in Figure 1) were well-balanced at entry and there were no significant differences regarding baseline parameters (Table 2). At baseline, 94% patients in the AP group and 89% patients in the placebo group (*P *= 0.31) fulfilled the criteria for septic shock (mean arterial pressure (MAP) ≤70 mmHg for at least one hour despite adequate fluid intake, or requirement for vasopressor support to maintain MAP). All patients were diagnosed with AKI according to the AKIN creatinine criteria or rise in serum creatinine level to > 150 μmol/L (1.70 mg/dL) within the previous 48 h. Furthermore, at baseline, there were eight anuric/oliguric (oliguria < 0.3 mL/Kg/h for > 6 h and serum urea > 20 mmol/L) patients in the placebo group (42%) and five anuric/oliguric patients in the AP group (31%, *P *= 0.14). One patient received study treatment (placebo) for less than one hour and had no efficacy evaluations beyond baseline. The decision to exclude this patient from the efficacy analyses was made blindly at the end of the trial, before code break. This patient was included in the safety analysis (conducted on all patients who took any study drug).

**Table 2:** Analysis sets and patients' characteristics at entry

Baseline Parameter*	AP (*n *= 16)	Placebo (*n *= 19)	*P *=
**Male: n (%)**	13 (81)	14 (74)	0.7003^†^
**Age: mean (SD) years**	65 (12)	67 (15)	0.7323^‡^
**Height: mean (SD) cm**	176 (10)	174 (8)	0.6275^‡^
**Weight: mean (SD) kg**	86 (12)	80 (14)	0.2207^‡^
**Heart rate: mean (SD) bpm**	103 (23)	105 (22)	0.8510^‡^
**Systolic BP: mean (SD) mmHg**	103 (26)	110 (26)	0.4140^‡^
**Diastolic BP: mean (SD) mmHg**	52 (13)	55 (13)	0.4035^‡^
**Temperature: mean (SD) °C**	37 (1)	37 (1)	0.5899^‡^
**APACHE-II score: mean (SD)**	24 (7)	23 (8)	0.5928^‡^
**SOFA score: mean (SD)**	10 (4)	11 (5)	0.9128^‡^
**AKIN stage 1: %**	44	58	0.0657^†^
**AKIN stage > 1: %**	56	42	
**Urine production: mean (SD) mL/kg/hour**	0.6 (0.4)	0.6 (0.7)	0.9245^‡^
**Serum creatinine: mean (SD) μmol/L**	164 (48)	214 (120)	0.1108^‡^
**Creatinine clearance: mean (SD) mL/minute**	50 (27)	40 (37)	0.3984^‡^
**(Nor)epinephrine: mean (SD; n) μg/kg/min**	0.32 (0.25;13)	0.40 (0.28;15)	0.3418‡
**Dopamine/dobutamine: mean (SD; n) μg/kg/min**	1.50 (2.12;2)	0.25 (0.35;2)	0.4975‡

* No significant differences between groups at baseline. ^†^Fishers's exact test and ^‡ ^t-test for two independent groups was used. All patients were Caucasians.

AKIN, Acute Kidney Injury Network; APACHE-II, Acute Physiology and Chronic Health Evaluation-II; CONSORT, Consolidated Standards of Reporting Trials; SD, standard deviation; SOFA, Sequential Organ Failure Assessment.

### Renal variables

**Figure 2:**
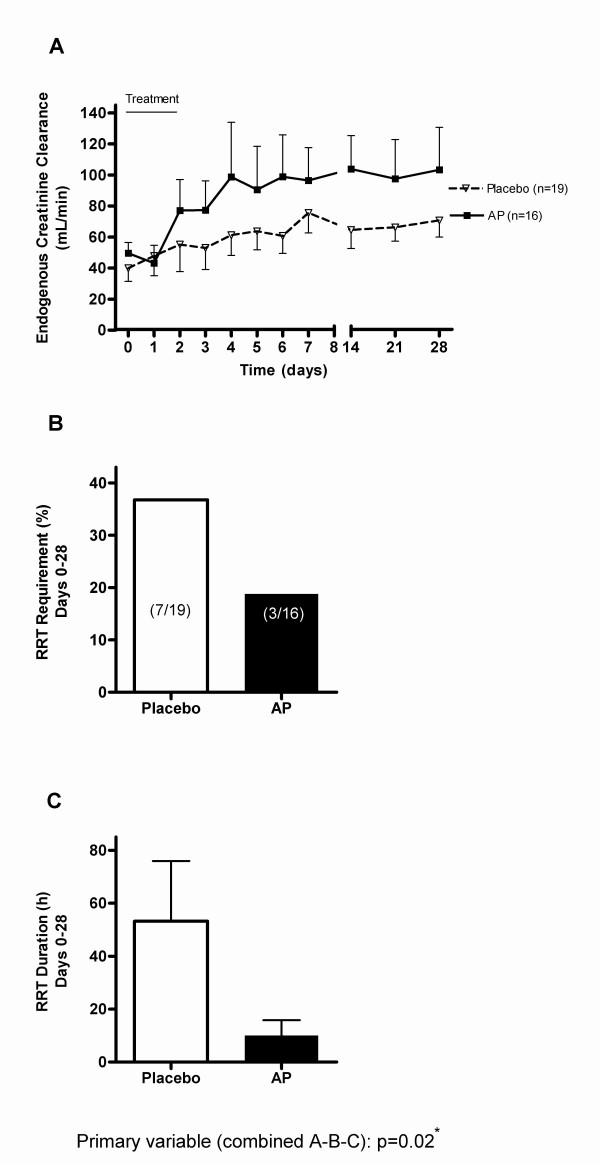
**Progression of renal parameters**. **(*A*) **Endogenous creatinine clearance is expressed as mean ± SEM (one-side depicted) and analyzed by ANOVA with repeated measurements over the complete curve (*P *= 0.01). Missing values were imputed from last-observation-carried forward (LOCF) from Day 7 to Day 28. **(*B) ***Renal replacement therapy (RRT) requirement is expressed as percentage in total treatment group; analyzed by Fisher's exact test (*P *= 0.29). **(*C*) **RRT duration: hours per total number of patients in group (cumulative for multiple interventions) over study period, expressed as mean ± SEM; analyzed by independent t-test (*P *= 0.08). *: Primary variable analyzed by the Hartung method [22].

The primary efficacy variable (renal parameters) showed a better outcome in the AP group (*P *= 0.02, Figure 2). The differences were maintained during exploratory analyses using other possible combinations of relevant renal parameters (see Additional file 1). Individually, the recovery of eCrCl was significantly more pronounced in the AP-treated group compared to the placebo-group during the first seven days, and this effect was sustained throughout the 28-day period (*P *= 0.01). In addition, regression analysis of eCrCl values excluding patients on RRT confirmed a significant (*P *< 0.03) and progressive effect of AP on clearance levels up to Day 7 post-entry (data not shown). Start of RRT, according to ADQI consensus criteria, was between one to three days after entry in all but two cases (one case in each treatment group). Patients who did not receive RRT were assigned zero days of RRT. RRT requirement was not different between both groups (Figure 1, *P *= 0.29), while RRT duration tended to be shorter in the AP group (*P *= 0.08). The effects of AP were similar in patients with or without proven Gram-negative infections (data not shown).

### Secondary efficacy parameters

#### Urinary biomarkers of renal injury

**Figure 3:**
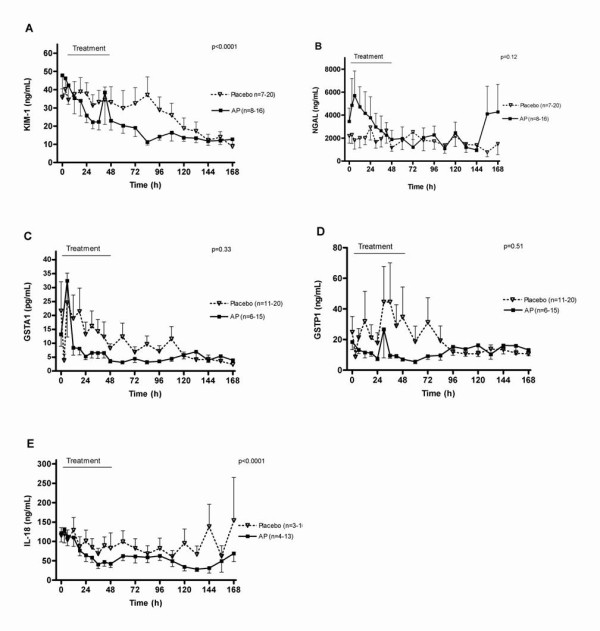
**Urinary biomarkers of renal injury**. **(*A*) **Kidney injury molecule-1 (KIM-1); (*B*) neutrophil gelatinase-associated lipocalin (NGAL); **(*C*) **glutathione S-transferase A1-1 (GSTA1-1); **(*D*) **GSTP1-1; and **(*E*) **IL-18 levels in urine; measured at various times points for placebo and alkaline phosphatase treatment during the first seven days. Urinary excretion of KIM-1 and IL-18 was lower in AP-treated patients relative to placebo-treated patients. Data are expressed as mean ± SEM (one-side depicted) and analyzed by ANOVA with repeated measurements over the complete curve with baseline as covariate.

During the course of the study, the decline of the urinary excretion of KIM-1 and IL-18 was significantly more pronounced in the AP group relative to placebo, while NGAL and GST enzymes were not significantly different (Figure 3).

#### Serum concentrations of inflammatory markers

**Figure 4:**
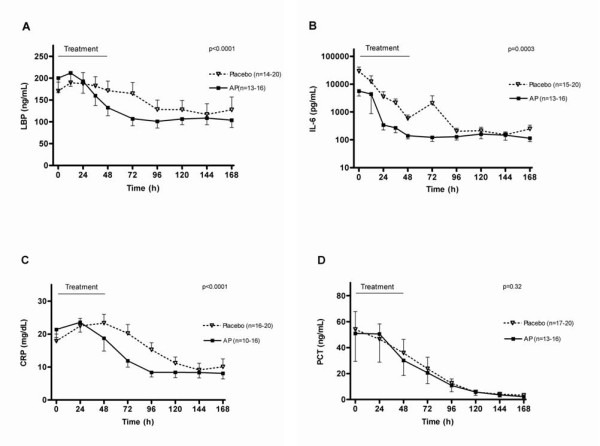
**Systemic inflammatory markers**. **(*A*) **LBP: Lipopolysaccharide-binding protein; **(*B*) **IL-6: interleukin-6; **(*C*) **CRP: C-reactive protein; **(*D*) **PCT: procalcitonin. Data are expressed as mean ± SEM (one-side depicted); analyzed by ANOVA with repeated measurements over the complete curve with baseline as covariate.

The decline in CRP, LBP and IL-6 levels was significantly more pronounced in the AP group, while PCT was not significantly different (Figure 4).

#### Clinical management parameters

Decrease in SOFA score evaluated during ICU stay was not significantly different between groups (see Additional file 3 for tabulated results of secondary clinical parameters). The reduction in total SOFA score was mainly caused by an improvement of kidney function. No relevant effects of AP on the other SOFA parameters were found. Length of stay in ICU was 25 ± 18 days for placebo and 11 ± 8 days for AP (*P *< 0.02). Total length of hospital stay was 31 ± 26 days on AP and 47 ± 36 days on placebo (*P *= 0.14).

The 28-day overall mortality after inclusion in the AP group was 7/16, compared with 6/20 in the placebo group (Log rank test, *P *= 0.25). All deaths were attributed to the underlying condition by the attending physicians and were subsequently reviewed by DSMB.

### Safety results

The incidence and type of AEs, serious and non-serious, were as expected for this population (Table 3). The incidence of treatment-emergent AEs was similar for the two treatment groups (AP: 124 events/16 patients; placebo: 147 events/20 patients). The independent DSMB did not raise any safety concerns during the trial.

**Table 3:** Safety results

		AP	Placebo
**All adverse events (AEs)**	n (%)	130 (100)	154 (100)
Treatment-emergent AEs*	n (%)	124 (95)	147 (96)
**Patients with treatment-emergent AEs**	n (%)	15/16 (94)	20/20 (100)
**Non-serious treatment-emergent AEs^a^:**	n		
Atrial fibrillation		3	6
Diarrhea		6	3
Hypotension		2	7
Delirium		2	5
Decubitus ulcer		1	4
Abdominal pain		3	2
Pyrexia		1	4
Impaired gastric emptying		3	2
Tracheostomy		1	3
Constipation		1	3
Restlessness		2	2
Atrial flutter		1	3
**All serious treatment-emergent AEs**	n		
Septic shock		2	2
Respiratory failure		3	1
Gastrointestinal necrosis		2	.
Hypotension		.	2
Hepatic necrosis		1	.
Gall bladder necrosis		1	.
Electrolyte imbalance		1	.
Azotemia		1	.
Cardiac arrest		.	1
Bradycardia		.	1
Electrocardiogram QT prolonged		.	1
Blood calcium decreased		.	1
Coma		.	1
Hyperlactacidemia		.	1
Depressed level of consciousness		.	1
Osteomyelitis		1	.
Brain neoplasm		.	1
Renal failure		.	1
Therapy cessation		1	.
Echocardiogram abnormal		1	.

AP, alkaline phosphatase.

*There were no treatment differences between the study groups regarding the number of Treatment-emergent AEs per patient (Fisher's exact test: *P *= 0.9089).

^a^: > 4 reported events in total.

## Discussion

In this prospective, randomized, double-blind, placebo-controlled phase-II trial in patients with severe sepsis or septic shock and evidence of AKI, administration of exogenous AP enzyme was shown to improve overall renal function, based on endogenous creatinine clearance, requirement and duration of renal replacement therapy. The latter were in line with urinary excretion of some markers of renal injury and with serum markers of systemic inflammation. By focusing our evaluations on patients with AKI secondary to sepsis, our results complement and expand our knowledge of the effects of AP previously reported in a general population of sepsis patients with and without AKI [14]. To the best of our knowledge, this is the first clinical trial in critically ill adults with sepsis that investigates measures of renal function combined with a panel of urinary biomarkers of renal injury. We found significant differences between treatment groups for KIM-1 and IL-18 excretion, both described to have prognostic importance for RRT requirement and mortality in patients with AKI [18,23]. Urinary excretion of other markers showed similar trends that did not reach significance likely due to a large variance between patients and inadequate power. For example, NGAL also showed a significant decrease in the AP-treated patients, while no change was observed in the placebo-treated patients. Because of the fact that the baseline values of NGAL were higher in the AP-group, this difference between groups did not reach significance. Apart from variance and the small patient groups, power was further attenuated by the fact that not all markers could be determined in all samples because of limited urine volume.

Animal experiments indicate that the dephosphorylating enzyme AP is depleted in the kidney following an ischemic insult [10]. Pharmacological restoration of endogenous AP levels in the kidney may prevent further renal damage or improve renal recovery, for which possible mechanisms include dephosphorylation of extra-cellular ATP. Extracellular ATP released from necrotic cells may directly activate the Nrlp3 inflammasome through the P2X_7 _receptor and co-stimulation by reactive oxygen species (ROS) or lipopolysaccharide (LPS) [24]. Alkaline phosphatase dephosphorylation of ATP and LPS results in reduced activation of the Nrlp3 inflammasome. As a consequence, the secretion of the pro-inflammatory cytokines is reduced, leading to less neutrophil infiltration in renal tissue, with lower local and systemic inflammatory response [25]. Dephosphorylation of extra-cellular ATP locally also results in higher levels of adenosine which, upon binding to its receptor, exerts potent anti-inflammatory and renal tissue protective effects [26,27]. The combined effect of modulation of local ATP and adenosine by AP results in reduced tissue damage, and the significantly faster reduction of the injury markers KIM-1 and IL-18 found in our trial corroborate this mechanism. Extracellular ATP levels are controlled by AP and other ectonucleotidases that are expressed along the renal arterioles and tubules [28]. The importance of ATP metabolizing enzymes is demonstrated by the finding that 5' ectonucleotidase (5'NT, or CD73) and NTDPase1 (CD39) provide protection against AKI in animal models [29,30]. The close structural and functional relationships between alkaline phosphatase and other ectonucleotidases and their therapeutic effect suggest that their modes of action may be similar.

Apart from local effects in AP-depleted kidneys, it is also thought that AP binds and detoxifies endotoxin that may be present in the blood stream [11], thereby modulating the inflammatory cascade in patients with sepsis, which may eventually result in less organ damage. We found a more pronounced decline in the systemic inflammatory parameters CRP, LBP and IL-6 which are known to be induced by endotoxin in patients treated with AP relative to controls [31]. This finding is in agreement with animal experiments [13], but was not found during experimental human endotoxemia using AP [32] or during the previous trial in patients with sepsis [14]. The large inter-individual variation in inflammatory markers during experimental human endotoxemia or in septic patients may account for this. In the present study we studied a more homogeneous group of severe sepsis or septic shock patients with AKI, while in the previous trial patients with sepsis with and without AKI were enrolled [14]. Since endotoxin is only present in Gram-negative bacteria and because of the putative relevance of AP-related detoxification of endotoxin, both completed phase-II trials in septic patients aimed to exclude Gram-positive sepsis. Nevertheless, almost half of enrolled patients did not show evidence of Gram-negative infections. Of interest, we found the beneficial renal effects of AP to be similar in patients with or without proven Gram-negative infections, but this does not exclude endotoxin detoxification being an important mechanism by which AP protects the kidneys. Indeed, in humans with severe sepsis or septic shock, increased circulating concentrations of endotoxin have been found in primary infections with both Gram-positive and Gram-negative bacteria [33]. Apparently, the primary infection is not a major determinant of increased circulating endotoxin levels in these patients, as intestinal translocation of endotoxin may also play a role [34]. In the present study we did not measure blood endotoxin levels, but we found that the kinetics of LBP was similar in patients with and without Gram-negative infections, and that the decline of LBP levels was significantly more pronounced in AP-treated patients. Thus, detoxification of endotoxin by AP may also contribute to the observed beneficial effects regardless of the primary bacterial pathogen. The observed effects of AP on the course of inflammatory biomarkers in the plasma suggest that AP exerts anti-inflammatory effects (possibly by detoxifying LPS); however, a direct effect of AP on the kidneys may also result in a swifter normalization of the circulating inflammatory biomarkers

There are several limitations of our study. First, the overall sample size was small. Availability of safe bovine AP enzyme and financial considerations were the main reasons for the small sample. The small number of adverse effects implicate that our study was not powered to detect differences in safety measures. Importantly, despite the latter and the heterogeneity of patients presenting with severe sepsis or septic shock, the remarkable consistency of findings in this trial (attenuation of urinary excretion of markers of renal damage and improved clinical outcomes), and in the previous trial in sepsis patients [14], suggest a strong signal that demands further study. The effects on eCrCl are likely to result in a beneficial effect on the need for RRT when tested in a larger trial. Nevertheless, we acknowledge that small differences in, for example, baseline creatinine may be responsible for some of the differences in outcome measures and this represents a limitation of the small study size. The enrollment criteria applied in this study may have resulted in inclusion of patients with AKI who were within their therapeutic window of opportunity. It is plausible that in previous studies [5,35] in which other, possibly effective, pharmacological interventions were tested in AKI, patients were enrolled in whom renal damage was beyond repair. The results of the two trials with bovine-origin AP enzyme and the restrictions of using bovine protein, indicate the need to develop a human recombinant AP and to evaluate its effects in clinical trials. Further trials should be conducted with caution and include sufficient interim analyses to determine if the AP treatment worsens mortality, irrespective of its impact on renal function.

A second issue that may need further explanation is the fact that we calculated the eCrCl for all patients, including those on RRT. We found that eCrCl was restored to normal range in the AP group within the first seven days, while it remained impaired in the placebo group. Although eCrCl calculations may overestimate the recovery of renal function in the non-steady-state period, the improvements in eCrCl were sustained during the whole study and remained significantly superior on AP treatment relative to placebo throughout the 28-day period. We are confident that the renal replacement therapy used in our study did not unduly or significantly influence eCrCl evaluations, and it was associated with stable, albeit lower serum creatinine values throughout the intervention period, although any such interference would have benefitted the control group which required proportionally more RRT interventions. Importantly, a sensitivity analysis excluding patients on RRT confirmed that CVVH did not significantly influence the interpretation of creatinine clearance data. Finally, one could argue that subjectivity of the chosen parameters for the primary efficacy variable of combined renal parameters (eCrCl, RRT requirement and duration) could influence the conclusions. For this reason, combinations of alternative parameters were tested exploratorily (see Additional file 1) and the beneficial effects of AP were shown to be maintained or further enhanced. The effects on eCrCl are likely to result in a beneficial effect on the need for RRT when tested in a larger trial.

## Conclusions

In septic patients with evidence of acute kidney injury, treatment with alkaline phosphatase improved overall renal function as represented by three main clinical parameters: endogenous creatinine clearance, requirement and duration of dialysis. The course of biomarkers of renal injury and systemic inflammation, as well as the clinical progression, corroborate the observed beneficial effects on renal function. These results suggest alkaline phosphatase treatment may be efficacious for these patients and a larger trial, preferably with recombinant human AP, is needed to further investigate these findings.

## Key messages

• Alkaline phosphatase is an endogenous enzyme that exerts detoxifying effects through dephosphorylation of endotoxins and pro-inflammatory extracellular ATP.

• Administration of bovine alkaline phosphatase to sepsis patients attenuates the urinary excretion of markers of tubular injury.

• In the present randomized, double-blind placebo-controlled phase 2 trial in patients with severe sepsis or septic shock with evidence of acute kidney injury treatment with alkaline phosphatase improved overall renal function as represented by three main clinical parameters: endogenous creatinine clearance, requirement and duration of dialysis.

• The results in renal parameters were supported by more pronounced reductions in circulating inflammatory markers and in the urinary excretion of markers of tubular injury in AP-treated patients relative to placebo.

## Abbreviations

5'NT: 5' ectonucleotidase; ADQI: Acute Dialysis Quality Initiative; AE: adverse event; AKI: acute kidney injury; AKIN: Acute Kidney Injury Network; AP: alkaline phosphatase; ATP: adenosine triphosphate; CRP: C-reactive protein; CV: coefficient of variation; CVVH: continuous veno-venous hemofiltration; DSMB: Data Safety Monitoring Board; eCrCl: endogenous creatinine clearance; ELISA: enzyme-linked immunosorbent assay; GSTA1-1: glutathione S-transferase A1-1; GSTP1-1; glutathione S-transferase P1-1; ICU: intensive care unit; IL: interleukin; ITT: intention-to-treat; KIM-1: kidney injury molecule-1; LBP: lipopolysaccharide-binding protein; LOCF: last-observation-carried forward; LPS: lipopolysaccharide; MAP: mean arterial pressure; MedDRA: Medical Dictionary for Regulatory Activities; NGAL: neutrophil gelatinase-associated lipocalin; PCT: procalcitonin; ROS: reactive oxygen species; RRT: renal replacement therapy; SAS: statistical analysis software; SOFA: sequential organ failure assessment.

## Competing interests

None of the authors has a financial relationship with a commercial entity that has an interest in the subject of this manuscript.

## Authors' contributions

PP wrote the manuscript and was responsible for patient enrollment, clinical management and manuscript review. SH conducted all ELISA assays for the study and wrote the manuscript. JS, PFL, JLV, AB, PJ and HS were responsible for patient enrollment, clinical management and manuscript review. MB planned and oversaw the statistical analysis. WP developed and supervised the ELISAs for GST measurements.

All authors have read and approved the manuscript for publication.

## Supplementary Material

Additional file 1 **Results of exploratory analyses of renal parameters **[37]. Click here for file

Additional file 2 **Methods for biomarkers of renal injury**. Click here for file

Additional file 3 **Results of secondary clinical parameters (non-renal)**. Click here for file
