# Supplementary material for: Alkaline phosphatase for treatment of sepsis-induced acute kidney injury: a prospective randomized double-blind placebo-controlled trial
Source: Crit Care. 2012 Jan 23;16(1):R14. doi: 10.1186/cc11159 (PMC3396250; doi:10.1186/cc11159)
Supplement: Additional file 1 — Results of exploratory analyses of renal parameters [37]. [file cc11159-S1.DOC]

**Additional file 1**

Manuscript by P Pickkers *et al*: ***Alkaline Phosphatase for Treatment of Sepsis-induced Acute Kidney Injury: A Prospective Randomized Double-Blind Placebo-Controlled Trial***

**Results of Exploratory Analyses of Renal Parameters**

Subjectivity of the chosen parameters for the primary efficacy variable of combined renal parameters (endogenous creatinine clearance (eCrCl), renal replacement therapy (RRT) requirement and duration) was tested exploratorily with possible combinations of the three chosen parameters with other available clinical renal parameters, namely (a) RRT relative duration, expressed as percentage of time on RRT relative to total length of time of each subject up to the end of study or death; (b) area-under-the-curve (AUC) of serum creatinine from baseline to end of study or death, and (c) regression analysis of eCrCl at days 0-7. The results of these exploratory analyses are provided in the additional file, 1 table 1.

**Additional file 1, Table 1: Exploratory analysis of renal parameters**

| **Combined renal parameters (primary variable)** * | **p =** |
| --- | --- |
| eCrCl + RRT requirement + RRT duration | 0.020 |
|  | |
| **Exploratory analyses of other combinations of renal parameters** * | |
| RRT requirement + RRT relative duration + eCrCl + AUC serum creatinine | 0.005 |
| RRT requirement + RRT relative duration + eCrCl | 0.018 |
| RRT total duration + eCrCl + AUC serum creatinine | 0.001 |
| RRT total duration + eCrCl | 0.016 |

LEGEND:

eCrCl: endogenous creatinine clearance; RRT: renal replacement therapy; AUC: area-under-the-curve.

For endogenous creatinine clearance, baseline-corrected repeated measures ANOVA over the complete curve (0-28 days) was used for the primary variable, and regression analysis (days 0-7) for exploratory analyses.

RRT Relative Duration: (RRT total duration)/(total study period; 28 days or death)x100.

*: Hartung method [37]
