# Supplementary material for: Alkaline phosphatase for treatment of sepsis-induced acute kidney injury: a prospective randomized double-blind placebo-controlled trial
Source: Crit Care. 2012 Jan 23;16(1):R14. doi: 10.1186/cc11159 (PMC3396250; doi:10.1186/cc11159)
Supplement: Additional file 2 — Methods for biomarkers of renal injury. [file cc11159-S2.DOC]

**Additional file 2**

Manuscript by P Pickkers *et al*: ***Alkaline Phosphatase for Treatment of Sepsis-induced Acute Kidney Injury: A Prospective Randomized Double-Blind Placebo-Controlled Trial***

**Methods for Biomarkers of Renal Injury**

**NGAL**

Neutrophil gelatinase-associated lipocalin (NGAL) was assayed by ELISA. The detection limit for NGAL was 0.315 ng/mL. All incubations were done in 100 μL/well and chemicals were of analytical grade and purchased from Sigma-Aldrich (Zwijndrecht, The Netherlands) or Merck (Darmstadt, Germany) unless stated otherwise. Capture antibody and detection antibody were obtained from R&D systems (Abingdon, UK). Capture antibody was diluted in 50 mM sodium carbonate buffer pH 9.6 (final concentration 1.97 μg/mL) and subsequently added to the wells of the Nunc Maxisorp 96-wells microtiter plates (Wiesbaden, Germany). After incubation for 16 hrs at 4°C, plates were washed three times (200 μL/well, ELx50, BioTek Instruments, Winooski, VT, USA) with phosphate-buffered saline (PBS; 140 mmol/L NaCl, 6.4 mmol/L Na_2_HPO_4_  and 1.3 mmol/L NaH_2_PO_4_, pH 7.4) supplemented with 0.1% (v/v) Tween-20 (Promega Corporation, Madison, USA). Urine samples were diluted 10, 100, or 1000 times in 0.1% (w/v) bovine serum albumin (BSA) in PBS. Calibration curves were made with purified NGAL, ranging from 0 to 20 ng/mL. Samples and calibrators were incubated for 1 hr at room temperature (RT). After three times washing, detection antibody diluted in 1% BSA (w/v) in PBS (final concentration 0.985 μg/ml) was added and incubated for 1 hr at RT. After washing, 0.5 μg/mL Streptavidin-horseradish peroxidase (Streptavidin-HRP, R&D, Minneapolis, USA) in 1% BSA in PBS was added and incubated for 20 min at RT. The plate was washed again and, subsequently, substrate solution, citrate-phosphate buffer pH 5 + 0.05% (w/v) O-phenylenediamine (OPD) + 0.012% (v/v) H_2_O_2_ was added and colour formation was stopped after 15 min at RT by adding 50 μl/well 0.5 M H_2_SO_4_.The absorbance was read at 490 nm in a Biotek plate reader (Powerwave 200, BioTek Instruments, Winooski, VT, USA)

**KIM-1**

Kidney injury marker-1 (KIM-1) was assayed by ELISA as described for NGAL. The detection limit for KIM-1 was 1.125 ng/mL. Capture antibody (final concentration 0.39 μg/ml in 50 mM sodium carbonate buffer, pH 9.6) and detection antibody (final concentration 0.39 μg/mL in 1% BSA in PBS, pH 7.4) were obtained from R&D systems. After incubation of the capture antibody during 1 hr at RT, Nunc Maxisorp plates were washed three times with PBS, pH 7.4 supplemented with 0.1% Tween-20 (PBS-T). Urine samples were diluted 2 or 10 times in 0.1% BSA in PBS. Calibration curves were made with purified KIM-1, ranging from 0 to 72 ng/mL. Finally, colour formation was stopped after 30 min at RT and absorbance was read at 490 nm in a Biotek plate reader.

**IL-18**

Interleukin-18 (IL-18) was determined semi-quantitatively. Nunc Maxisorp plates were coated with 125 ng/mL IL-18 antigen (Randox Life Sciences, Crumlin, UK) diluted in sodium carbonate buffer pH 9.6 and incubated 16 hrs at 4°C. Undiluted urine samples (60 µL) were added to wells of a roundbottem Greiner plate (Greiner Bio One, Alphen a/d Rijn, The Netherlands) and 60 µl binding antibody (sheep anti human IL-18 antibody, Randox) was added to the samples (1 μg/mL final concentration in 1% BSA in PBS) and incubated 16 hrs at 4°C. Plates were washed and 100 µL of the sample/antibody mixture was added to the wells of the Maxisorp plates. Calibration curves were made with purified IL-18, ranging from 0 to 250 pg/mL. After three times washing, Rabbit anti Sheep HRP-labeled antibody, diluted in 1% BSA in PBS was added and incubated for 1 hr at RT. Finally, colour formation was stopped after 45 min at RT and absorbance was read at 490 nm in a Biotek plate reader.

**GST-A1-1**

Immediately following collection, urine for both the determination of glutathione S-transferase alpha 1-1 (GST-A1-1) and GST-P1-1 was stored in a 10% (v/v) buffer solution, pH 7.5, containing 1 M HEPES, 5% (w/v) BSA, 1% (w/v) sodium azide, 1% (v/v) Tween-20 (Promega Corporation) and 10% (v/v) glycerol to ascertain a stable pH, to prevent bacterial growth and to prevent binding of GSTs to the tubes.

GST-A1-1 was assayed by ELISA and the detection limit was 0.781 ng/mL. Polystyrene microtiter plates (Greiner) were coated during 16 hrs at 4°C with 10 μL ascites/500 μL PBS (pH 7.4) purified anti-GST-A1-1 monoclonal antibody 5B11 2F1 and blocked for 1 hr with 200 μl/well PBS supplemented with 1.25% (v/v) Tween-20 and 1% (w/v) BSA (PBS-T-BSA, pH 7.4) at RT. Urine samples were diluted 2, 10, or 20 times in filtrated PBS-T, pH 7.4, with 20 mmol/L ethylenediaminetetraacetic acid (EDTA) and 10% (v/v) normal human plasma (NHP; supernatant from heat-treated plasma at 60°C for 60 min). Calibration curves were made in duplicate with purified GST-A1-1, ranging from 0 to 50 ng/ml, diluted in PBS-T-EDTA-NHP. Samples and calibrators were added to the wells of the microtiter plates and incubated overnight at RT. Plates were washed (200 μL/well) three times with PBS supplemented with 1.25% Tween-20 and subsequently incubated with rabbit anti-GST-alpha antiserum diluted 1:4000 in PBS-T-BSA for 3 hrs at RT. Plates were washed and incubated for 2 hrs with peroxidase-labeled swine anti-rabbit antibody (Dakocytomation, Glostrup, Denmark) diluted 1:2000 in PBS-T-BSA. Plates were washed five times and 3.75 μmol/L OPD, 0.04% (v/v) H_2_O_2_ in 24 mmol/L sodium citrate and 51 mmol/L Na_2_HPO_4_, pH 5 was added to the wells. After 30 min incubation, the reaction was stopped by adding 100 μL 4N H_2_SO_4_ and absorbance was read at 492 nm with a background subtraction at 620 nm.

**GST-P1-1**

Glutathione S-transferase P1-1 (GST-P1-1) was assayed by ELISA as described for GST-A1-1. The detection limit for GST-P1-1 was 1,563 ng/mL. Polystyrene microtiter plates (Greiner) were coated during 16 hrs with at 4°C with 10 μL ascites/500 μL PBS (pH 7.4) purified anti-GST-P1-1 monoclonal antibody and blocked for 1 hr with 200 μl/well PBS-T-1% BSA (pH 7.4) at RT. Urine samples were diluted 2, 10, or 20 times in filtrated PBS-T-EDTA-10%NHP and calibration curves were made in duplicate with purified GST-P1-1, ranging from 0 to 100 ng/mL, diluted in PBS-T-EDTA-10%NHP. After overnight incubation at RT, plates were washed and subsequently incubated with rabbit anti-GST-P1-1 antiserum diluted 1:1000 in PBS-T supplemented with 10% NHP for 3 hrs at RT. Finally, the detection and staining was similar as described above for GST-A1-1 and absorbance was read at 492nm with a background subtraction at 620 nm.
