# Supplementary material for: Alkaline phosphatase for treatment of sepsis-induced acute kidney injury: a prospective randomized double-blind placebo-controlled trial
Source: Crit Care. 2012 Jan 23;16(1):R14. doi: 10.1186/cc11159 (PMC3396250; doi:10.1186/cc11159)
Supplement: Additional file 3 — Results of secondary clinical parameters (non-renal). [file cc11159-S3.DOC]

**Additional file 3**

Manuscript by P Pickkers *et al*: ***Alkaline Phosphatase for Treatment of Sepsis-induced Acute Kidney Injury: A Prospective Randomized Double-Blind Placebo-Controlled Trial***

**Results of Secondary Clinical Parameters (non-renal)**

Secondary clinical endpoints (length of ICU and hospital stay, duration of ventilator support and changes in SOFA scores) are displayed in the table below.

**Additional file 3, Table 1: Secondary Clinical Parameters (non-renal)**

| **Non-renal Clinical Endpoints** | **Placebo** | **AP** | **p** |
| --- | --- | --- | --- |
| Length ICU stay: days, mean (SD) | 25 (18) | 11 (8) | <0.02^†^ |
| Length hospital stay: days, mean (SD) | 47 (36) | 31 (26) | 0.23^†^ |
| Length of ventilator support: days, median (95% CI) | 21 (4-26) | 5 (4-29) | 0.66* |
| SOFA Score change 0-7 days: mean (SD) | 4 (3) | 6 (3) | 0.20^†^ |

LEGEND:

ICU: intensive care unit; SOFA: sequential organ failure assessment score; CI: Confidence interval.

^†^: t-test; *: log-rank
